# Improving TMJ Diagnosis: A Deep Learning Approach for Detecting Mandibular Condyle Bone Changes

**DOI:** 10.3390/diagnostics15081022

**Published:** 2025-04-17

**Authors:** Kader Azlağ Pekince, Adem Pekince, Buse Yaren Kazangirler

**Affiliations:** 1 Department of Oral and Maxillofacial Radiology, Karabuk University, Karabuk 78600, Turkey; azlagkader@karabuk.edu.tr; 2 Department of Computer Engineering, Karabuk University, Karabuk 78600, Turkey; buseyaren@uky.edu; 3 Department of Internal Medicine, University of Kentucky, Lexington, KY 40506, USA

**Keywords:** temporomandibular joint, mandibular condyle, degenerative bone changes, deep learning, convolutional neural networks, panoramic radiography

## Abstract

**Objectives**: This paper evaluates the potential of using deep learning approaches for the detection of degenerative bone changes in the mandibular condyle. The aim of this study is to enable the detection and diagnosis of mandibular condyle degenerations, which are difficult to observe and diagnose on panoramic radiographs, using deep learning methods. **Methods**: A total of 3875 condylar images were obtained from panoramic radiographs. Condylar bone changes were represented by flattening, osteophyte, and erosion, and images in which two or more of these changes were observed were labeled as “other”. Due to the limited number of images containing osteophytes and erosion, two approaches were used. In the first approach, images containing osteophytes and erosion were combined into the “other” group, resulting in three groups: normal, flattening, and deformation (“deformation” encompasses the “other” group, together with osteophyte and erosion). In the second approach, images containing osteophytes and erosion were completely excluded, resulting in three groups: normal, flattening, and other. The study utilizes a range of advanced deep learning algorithms, including Dense Networks, Residual Networks, VGG Networks, and Google Networks, which are pre-trained with transfer learning techniques. Model performance was evaluated using datasets with different distributions, specifically 70:30 and 80:20 training-test splits. **Results**: The GoogleNet architecture achieved the highest accuracy. Specifically, with the 80:20 split of the normal-flattening-deformation dataset and the Adamax optimizer, an accuracy of 95.23% was achieved. The results demonstrate that CNN-based methods are highly successful in determining mandibular condyle bone changes. **Conclusions**: This study demonstrates the potential of deep learning, particularly CNNs, for the accurate and efficient detection of TMJ-related condylar bone changes from panoramic radiographs. This approach could assist clinicians in identifying patients requiring further intervention. Future research may involve using cross-sectional imaging methods and training the right and left condyles together to potentially increase the success rate. This approach has the potential to improve the early detection of TMJ-related condylar bone changes, enabling timely referrals and potentially preventing disease progression.

## 1. Introduction

The temporomandibular joint (TMJ) is a pair of joints formed by the articulation of the mandibular bone with the temporal bones, located symmetrically on both sides of the head. The space between the condylar process of the mandibular bone and the articular fossa of the temporal bone is divided into upper and lower joint compartments by the articular disc. This joint is supported by ligaments.

Due to its anatomical structure, the temporomandibular joint can move in three planes. This allows the physiological movements of speaking, chewing and swallowing to be easily performed. These physiological movements are made possible by the combination of the elevation–depression, protrusion–retraction and lateral translation movements [1,2] of the TMJ. The TMJs are connected via the mandible, which adds to the complexity of these movements. Although each TMJ is an independent joint [3,4], they move together and affect each other’s direction and range of motion.

Exceeding the physiological limits of joint movements, infections, trauma, or biological factors can cause pain and dysfunction in the chewing muscles and TMJ. This condition is called temporomandibular disorder (TMD). TMD can progress from disc dysfunction to osteoarthritis, but this outcome is not always certain. In cases where osteoarthritis does develop, radiographic findings are often seen in the mandibular condyle and articular eminence [5].

TMD is a common health problem that significantly reduces quality of life, affecting an average of 34% (Asia—33%, South America—47%, North America—26%, Europe—29%) of the world’s population according to a meta-analysis study [6]. Okeson has classified TMDs into muscle-related disorders, disc displacements, inflammatory joint diseases, and genetic and acquired anatomical abnormalities. This classification demonstrates that TMD encompasses a broad spectrum of diverse pathologies and can manifest with a variety of symptoms.

Temporomandibular joint osteoarthritis, an inflammatory joint disease and a subtype of TMD, is one of the most frequently observed degenerative joint disorders. Osteoarthritis is a painful inflammatory condition. Osteoarthritis is defined by clinical symptoms and radiological signs [7]. It causes changes in the joint surfaces and arises as a consequence of disc displacement, trauma, functional overload, and developmental anomalies [8]. These factors contribute to the joint components being loaded beyond their adaptive capacity, and in some instances, to their exposure to prolonged and destructive loads [9]. This scenario initiates a physiological process characterized by degenerative changes in the bones, aimed at accommodating the joint surfaces to the functional demands [10]. As these processes persist and advance, morphological alterations manifest in the bones. Such alterations are more prevalent in the mandibular condyle than in the glenoid fossa or articular eminence [11]. Degenerative bone changes occurring in the TMJ can be observed as erosion, osteophyte formation, sclerosis and subcortical cyst formation in the mandibular condyle [8,12].

Often the arthritic condition can become adaptive once the load is reduced, but the bony morphology remains altered. The adaptive stage is known as osteoarthrosis.

Osteoarthritis and osteoarthrosis are classified as degenerative joint diseases (DJD) of the temporomandibular joint [5].

Meta-analysis findings revealed that TMJ DJD is observed in 10% of the general adult population and ranges from 18% to 85% among patients with TMD [13]. Furthermore, a study of elderly people with TMD found that 70% of elderly people had bone changes in their temporomandibular joints, and 69.93% of these changes were seen in the condyles [14].

The multifactorial etiology of TMD leads to a diversity of treatment approaches. In some cases, TMD treatment requires an interdisciplinary approach involving collaboration among specialists. Correct and early diagnosis of this condition, which affects a large segment of society, is critical for determining appropriate treatment strategies and managing the disease.

In some studies conducted to measure the level of knowledge of general dentists about TMD, it was concluded that the level of knowledge of general dentists was inadequate and would be insufficient to provide effective care to patients with TMD [15,16].

Artificial Intelligence (AI) is now widely used in every field, bringing convenience and practicality to every area where it is applied. With the increase in workload and time requirements, AI has become very useful. Today, the application of AI and especially computer vision techniques in the medical field is becoming much more widespread [17]. For this reason, a decision support mechanism that will receive support from AI rather than a determination based on human power is often a preferred solution [18].

Artificial Intelligence (AI), including the development of machine learning tools and neural networks, has developed rapidly over the last decade. Various medical applications have been developed with the support of AI to save clinicians’ time during examinations and to demonstrate the ability to make more objective diagnoses [19,20]. Neural network cells used for AI are a type of network that makes up artificial neural networks. In particular, Convolutional Neural Networks (CNN), a type of artificial neural network, are used in detection and image classification studies for image data. Medical imaging technology plays a vital role in several critical applications, from early detection of diseases to surgical planning. Innovative approaches in this field include medical image classification and detection, allowing for faster and more accurate detection of diseases. While traditional classification methods are often inadequate for this complex task, deep learning techniques, especially CNN, have revolutionized this field. These algorithms used by AI can analyze large amounts of clinical data on different diseases and consequently aim to minimize diagnostic and treatment errors that are common in standard clinical settings [21]. CNN are deep learning algorithms for 2D data that take input images and perform convolution with filters or kernels to extract features. Moreover, CNN networks have been proven to outperform experts in many image detection studies and tasks [22]. In particular, CNN algorithms have been chosen for image classification and interpretation, considering that CNN algorithms are a powerful and effective choice in common literature studies of researchers. Thus, in this study, deep learning techniques were developed to make decisions about the clinical condition of patients. As a result, CNNs offer an innovative approach to medical image classification, providing healthcare professionals with powerful applications for more accurate diagnosis and treatment planning. This paper will explore the potential of CNN-based approaches in detecting different condyle degenerative bone changes in TMJ.

### Transfer Learning with Convolutional Neural Networks

One of the main reasons why CNNs are especially preferred in medical image classification is the ability to emphasize local features and extract hierarchical features thanks to convolutional layers [9,23]. Transfer learning is a strategy that allows knowledge learned in one task to be used more quickly and effectively in another task. CNN-based transfer learning can improve the model’s performance when working with limited datasets in image classification [24]. Moreover, by transferring knowledge from a general dataset to a specialized dataset, transfer learning allows for more specific results in a specific medical application area. Therefore, using transfer learning for CNN networks saves time by avoiding the need to re-learn attributes and weights. Thus, it offers the potential to overcome dataset limitations during the model’s training and learning phase and increase its generalization capability [25]. Farook et al. [26], who presented a comprehensive review study for the clinical classification of degenerative disorders in TMJ, presented many approaches to traditional diagnostics obtained from radiographs while addressing the causes of deformations and included studies in the literature in detail. According to the study’s results, neural network models learned through deep learning were found to diagnose the detection in 2D or 3D radiographs as accurately as clinicians. According to the research mentioned in the study (depending on the dataset used, types of deformation, etc.), the top-performing deep learning algorithms are usually pretrained algorithms such as Random Forest (RF), Multi-Layer Perceptron (MLP), AlexNet, Support Vector Machine (SVM), Extreme Gradient Boost (XGBoost), LightGBM, Residual Network (ResNet), Visual Geometry Group Network (VGGNet), etc. However, algorithms such as SVM, RF, XGBoost, LightGBM, etc., are frequently used in machine learning and leave feature extraction to the user [27].

Deep learning has revolutionized medical image analysis by enabling more precise and efficient diagnostic processes. This study aims to leverage deep learning techniques, particularly CNNs to enhance the accuracy and efficiency of medical image classification in detecting degenerative bone changes in the TMJ. The selection of pre-trained CNN architectures was guided by their proven effectiveness in medical image classification tasks and their architectural diversity. The purpose of this was to facilitate a comprehensive performance comparison of the models. The models selected for the study were AlexNet, VGG16, VGG19, ResNeXt (18, 101), DenseNet (121, 169, 201), ResNeXt and GoogleNet. It is important to note that these models represent different network depths, connectivity models and parameter efficiencies. The prevailing trend in the field of healthcare towards a greater reliance on AI-driven decision support systems is the focus of this research. The potential of CNN-based models, including pretrained architectures such as AlexNet, VGGNet, ResNet, and DenseNet, to improve diagnostic precision will be explored. Furthermore, transfer learning strategies will be employed to optimize model performance on limited medical imaging data. The integration of cutting-edge deep learning methodologies is a key aspect of this study, with the aim of contributing to the advancement of automated diagnostic tools. These tools are expected to reduce human error and provide clinicians with more objective and reliable assessments. This study uses various proposed CNN architectures to improve the corresponding system performance while keeping the underlying learning topologies constant. In this study, the most widely used pretrained CNN algorithms are AlexNet, VGG16 and VGG19, DenseNet121, DenseNet169, DenseNet201, ResNet18, ResNet101, ResNeXt, and GoogleNet. These algorithms are famous CNN architectures introduced for object recognition and classification tasks [25,28,29].

Several studies have used different radiological methods and classification systems to evaluate temporomandibular joint osteoarthritis. One study [30] examined 3514 cone-beam computed tomography (CBCT) images from 314 patients and classified changes in condylar structure were divided into three groups: no evidence of TMJOA, indeterminate for TMJOA, and evidence of TMJOA. Another study [31] using panoramic radiographs used the same classification system. A separate study [32] of 858 panoramic radiographs classified the presence of osteoarthritis as “osteoarthritis” or “normal”.

Bony changes observed in the mandibular condyle may show different variations, ranging from erosion to deformation of the condyle morphology. A detailed classification of these findings provides the clinician with information about the status of the disease.

Given the progressive nature of TMD, early detection of osseous alterations in the mandibular condyle, irrespective of etiology, is important.

Because patients frequently present to general dental practitioners as their initial point of contact, and given the constraints faced by these practitioners in accessing and interpreting CBCT, the significance of panoramic imaging in detecting bone changes is increasing.

Panoramic radiography, which is widely used in routine clinical practice, has the potential to contribute to the early diagnosis of TMD by allowing the evaluation of the temporomandibular joint. Based on previous research, this study aims to comprehensively evaluate the potential of deep learning in detecting and classifying degenerative bone changes of the mandibular condyle on panoramic radiographs through comparative analysis of multiple CNN architectures on a large dataset.

## 2. Materials and Methods

### 2.1. Selection, Preparation and Evaluation of Images

For the evaluation of mandibular condyle bone changes, 2300 randomly selected panoramic radiographic images in DICOM format taken between January 2023 and September 2023 from the X-ray archive of Karabük Oral and Dental Health Training and Research Hospital were examined. These images were obtained using the same panoramic radiography device (I-Max touch, Owandy Radiology, Croissy-Beaubourg, France) in accordance with the manufacturer’s instructions (80 kV tube voltage, 9 mA and 14.4 s).

### 2.2. Dataset and Preprocessing Steps

Of the panoramic radiographic images examined by the oromaxillofacial radiology specialist, a total of 725 condylar images were excluded from the study, including 248 with superposition that impaired the observation of condylar borders, 189 with artifacts, and 288 images from 144 individuals under the age of 18. Given that age and sex are not matched in the condyle, all models and results presented in this study could be sex and gender biased. Further research is required to address this limitation in future studies with balanced data. Furthermore, all experiments were performed in a subject-independent manner in the study, meaning that images from the same subject were not used in the training and testing phases. A total of 3875 condylar images from individuals over the age of 18, in which the mandibular condyles could be clearly observed, without artifacts, were included in the study. The study included a total of 3875 subjects with 1 image per subject.

The borders of the mandibular condyle are normally straight, continuous, and convex. Therefore, it is assumed that the outlines of normal condyles should have a convex configuration everywhere and that there should be symmetry between both condyles in the same individual [33]. However, anatomical variations such as slight flattening or pronounced convexity on the upper surface of the condylar head may be seen. In this case, the size and shape of the right and left condylar heads should be compared and the symmetry of both sides should be evaluated [34].

Another preprocessing step is to make the data compatible with CNN algorithms. CNN algorithms typically work in 224 × 224 dimensions. These dimensions may increase and decrease depending on the working structure of CNNs and the feature mapping technique. The images were automatically cropped for the CNN architectures used, and the input data were subjected to specific preprocessing steps to normalize them [21]. This will help the network improve learning performance because transfer learning will be provided with previously learned weights. For the standardization process, normalization was performed using mean values [0.485, 0.456, 0.406] and standard deviation values [0.229, 0.224, 0.225] [22].

In our study, condyles with regular convex and continuous borders were evaluated together with their symmetry and labeled as normal. Condyles that did not meet these criteria, with interrupted borders, straight but not symmetrical or concave borders, were included in the other class. Condyles with mild flattening or significant convexity were included in the normal class when they were symmetrical and were included in the other class when they were unilateral. All condyles with osteoarthritic changes such as erosion and osteophyte formation, other than significant flattening, were labeled as other. Images in which the convexity at the outer borders of the mandibular condyles was disrupted in favor of flattening were labeled as flattening. Images in which the continuity of the outer borders of the condyle was disrupted were labeled as erosion.

In addition, while evaluating condylar bone changes in our study, flattening, osteophyte, erosion and images in which two or more of these changes were observed together were classified as the other group. Images in which condyles with pseudocysts were observed were not evaluated as a separate class because they did not affect the outer borders of the mandibular condyle observed on the panoramic images.

Classifications were made for the study. Since each group did not contain enough images for computer learning, two separate approaches were made for the images belonging to the condyles with erosion and osteophyte formation. In the first approach, osteophyte formation and erosion groups were included in the other group and the images were collected in three groups: normal, flattening and deformation. In the second approach, osteophyte and erosion groups were removed from the study and grouped as normal, flattening and other. The deformation group became a more comprehensive group compared to the other group.

The mandibular condyles were resized to 224 × 224 (width and height) dimensions to be compatible as input to CNN models. Nonetheless, there was an absence of coordinate labeling of the condyle images. Instead, the mandibular condyles were categorized into clusters by an oromaxillofacial radiologist. This approach is informed by the nature of the study, which is an image classification task. To ensure optimal feature extraction and to focus on clinically relevant areas, a preprocessing step was implemented before feeding images into CNN architectures. Since CNN models require a fixed input size, the following methodology was applied:

Region of Interest (ROI) Selection: Rather than employing full panoramic radiographs, which comprise a large amount of extraneous background information, radiologists manually identified the mandibular condyle regions in the images. The ROIs were then cropped so as to isolate the condylar region. This was performed in order to ensure that the CNN models focus on the most relevant anatomical structures.

Automated Resizing to Proper Sizes: Following the cropping of the ROI, the images were resized to 224 × 224 pixels by means of bilinear interpolation. This step is of paramount importance as it ensures compatibility with pretrained CNN models while preserving critical features necessary for classification. This approach has been demonstrated to minimize irrelevant variations in input images, thereby improving the data of model focus on clinically significant patterns and optimizing the learning process. A randomly selected 25% of the labelled images were re-examined by the same clinician at a different time to assess intra-observer agreement. The agreement of the results was assessed using Cohen’s kappa analysis. SPSS (Statistical Package for Social Sciences) for Windows 20.0 was used for the Kappa test. When the obtained κ value (κ = 0.8) was interpreted according to Landis and Koch, it was found that there was sufficient agreement.

**Figure 1:**
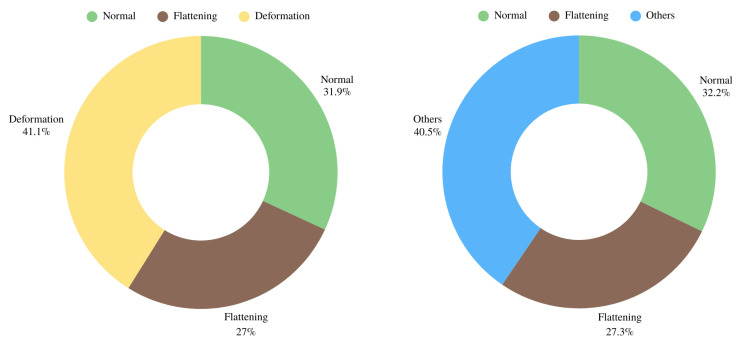
Weighted scatter plot of prepared data classes including normal-flattening-deformation and normal-flattening-others classes.

Mandibular condyle changes: mandibular condyle regions in panoramic radiographs were cropped and labeled by the oromaxillofacial radiology specialist. Mandibular condyle changes and regions in panoramic radiographs were cropped and labeled. Classifications were made for the study. Since each group did not contain enough images for computer learning, two separate approaches were made for condyles with erosion and osteophyte formation images. In the first approach, osteophyte formation and erosion groups were included in the other group, and the images were collected in three groups: normal, flattening, and deformation (N,F,D). The second approach removed osteophytes and erosion groups from the study and grouped them into normal, flattening, and other (N,F,O) groups. The deformation group became a more comprehensive group compared to the other group. A deep learning-based algorithm approach was applied for high-performance detection and classification of condyle bone changes in cropped images as normal, flattening, and other (N,F,O) and normal, flattening, and deformation (N,F,D). The study applied two different datasets to CNN architecture, and their successes were discussed. To develop the Artificial Intelligence (AI) approach, two different sets will be separated into different sets for the training and testing process. In AI applications, randomness should be used as a basis for applying the data to the training and testing process. The set is divided into 70:30 and 80:20 training tests, respectively, as shown in Figure 1. In the experimental findings section of the study, this separated set will reveal the differences in the success of CNN algorithms. Different dataset separations are applied to solve the balance problem.

In AI applications, randomness should be taken as a basis for applying the data to the training and testing process. The set was split into 70:30 and 80:20 training–testing, respectively, as shown in Figure 1. The experimental findings section of the study will reveal the differences in the success of the CNN algorithms. The different separations of the data were applied to solve the balance problem.

**Figure 2:**
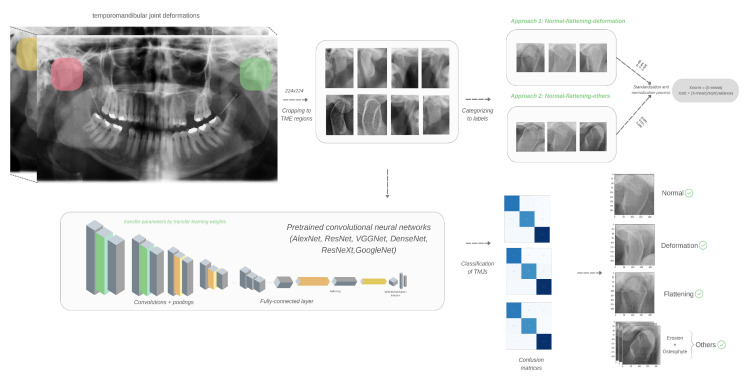
Different CNN architectures are proposed for the detection of morphologies caused by degenerative bone changes in temporomandibular joints in panoramic images.

The processing step was applied separately for training and testing steps Figure 2 is an architectural visualization representing the working structure of the different CNN architectures proposed for detecting degenerative bone changes in the temporomandibular joints in panoramic images. First, the size of the input images from the TMJ region should be standardized for all instances. The figure shows that this standard form is set to 224 × 224 for the example CNNs. For this reason, the TMJ regions cropped by the oromaxillofacial radiology specialist are scaled to 224 × 224 by a preprocessing step before being fed to the model. Many different architectures were used in this study to access the experimental findings for the pretrained CNN algorithms with version 1.13.1 of the PyTorch library. Therefore, each architecture contains different layers, such as convolution, pooling, etc., in its internal structure. For this reason, a typical internal architecture structure is depicted in Figure 2.

In the present study, a range of pretrained architectures was utilized to assess the efficacy of models. The input data are constituted by an image, upon which sequential mathematical operations are applied in the context of pretrained CNN algorithms. In CNN models, these parameters comprise convolution kernels (filters), weights, and biases of the fully connected layers. For fully connected layers within CNNs, refer to the following sources: the linear transformation is modeled as W weight matrix, b bias term, and X input vector in Equation (1).

Y = WX + b(1)

The image X matrix as an input vector denotes the value of each pixel and typically assumes the form of a 3D tensor (height, width, number of channels. In this context, the prediction function of the model is denoted by y^ = f(X,θ). According to the specified equation, X denotes the input data received by the model. The class prediction function is usually expressed as y^ is the class or probability distribution predicted by the model. In the context of an image-based AI model, X corresponds to an image matrix. Conversely, θ encompasses the weights and bias values that the model must learn. Furthermore, the Rectified Linear Unit (ReLU) function, a widely utilized activation function, is formulated in Equation (2).

ReLU(x) = max(0,x)(2)

Accordingly, x represents the weighted sum, and the output of the neuron is calculated by inserting x into the ReLU activation function. As the model undergoes training, the optimization algorithm updates these parameters to ensure the most accurate prediction. During the training process, the categorical cross entropy loss function is employed to minimize the classification error. The optimization process involved the update of model weights using the SGD and Adamax algorithms. In addition, the Adamax algorithm has been shown to outperform the SGD algorithm in other models. The formulas for mean moment estimation, infinite norm estimation, and parameter update are given in Equations (3), (4), and (5), respectively. It is noteworthy that this algorithm utilizes infinite-norm estimation; consequently, it provides more balanced updates when the weights are substantial. According to these equations, β_1_ is the momentum term for the first-moment estimate, g_t_ is gradient time step t, and also m_t_ is the first-moment vector. On the other hand, β_2_ refers to the decay rate for the exponentially weighted infinity norm, u_t_ is an infinity norm (maximum absolute value of past gradients), Θ_t_ model parameter at time step t, and also α is a learning rate.

m_t_ = β_1_ × m_t_ − 1 + (1 − β_1_) × g_t_ (3)

u_t_ = max(β_2_ × u_t_ − 1, |g_t_|) (4)

Θ_t+1_ = Θ_t_ − ((α/u_t_) × m_t_) (5)

Figure 2 shows a typical internal architecture. The automatically cropped data are organized into folders according to the selected study. For example, for approach 1, normal, flattening, deformation; for approach 2, normal, flattening, others. Both datasets were subjected to the standardization above and normalization for preprocessing. The convolution layers, especially the first convolution layer from which the input is taken, use multiple filters to capture various edge and texture information. Then, the activation function is appropriate for the chosen CNN algorithm. The pooling layer follows to reduce the dimensionality and preserve important features. Multiple convolutions and pooling layers are added to increase the depth. This enables capturing more complex features (e.g., detailed characteristics of degenerative changes). After the convolution and pooling layers, the resulting feature map is smoothed and fed into multiple fully connected layers to reach the output layer. In the output layer, the appropriate activation function is designed to classify different condyle bone changes, and the classification process is completed depending on the number of classes. Furthermore, CNNs were trained using the Adam optimizer with a learning rate of 0.001 to provide adaptive learning adjustments to enhance convergence. To enhance stability during the training process, a step learning rate scheduler was employed, which reduced the learning rate by a factor of 0.1 every 10 epochs. This approach prevented overshooting and ensured gradual refinement of the model’s parameters. The training process was conducted for 200 epochs, enabling the model to acquire robust feature representations. The loss function employed was categorical cross-entropy, a well-suited choice for multiclass classification problems. The hyperparameter choices were made to balance training efficiency and classification accuracy while ensuring optimal model performance.

## 3. Results

In this study, the mandibular condyle regions are cropped on panoramic radiographs, and the approach of artificial intelligence-based algorithms is applied to classify the condyle bone changes into normal, flattening, deformation, and variants belonging to the normal, flattening, and other categories for high-performance detection. To achieve the main objective of the study, which is to detect different condyle degenerative bone changes in the temporomandibular joint most successfully, various datasets belonging to different distributions were used and evaluated with different learning techniques. Many different pretrained CNN algorithms were applied in the study, and their experimental findings are given in Table 1 and Table 2. For the experimental findings in the tables, the accuracy, precision, recall, and F1-score metrics in Equations (6)–(9), which are frequently used in object detection and classification studies, were used. These metrics capture not only the accuracy performance of the model but also the miss rates and successes of negative and positive classes. In particular, the F1-score metric provides an overall view by obtaining the harmonic mean of the precision and recall metrics [35]. Thus, it proved why CNN algorithms are preferred for convolutional filtering and classification of health images.

(6) $$Accuracy=\frac{TP+TN\;}{TP+FP+TN+FN}$$

(7) $$Precision=\frac{TP\;}{TP+FP}$$

(8) $$Recall=\frac{TP\;}{TP+FN}$$

(9) $$F1-Score=2\times \frac{Precision\times Recall}{Precision+Recall}$$

Table 1 presents simple statistical analyses for a single dataset (N, F, O) with different CNN algorithms. Experiments were performed on equal terms at this stage when using multiple pretrained neural networks. At the beginning of the experiments, the learning rate was chosen as 0.001, but it was found that the results were more successful with a learning rate of 0.0001. For this reason, in the experimental findings, only the best results (*lr* = 0.001) for DenseNet121 are included, while the results for the other algorithms (*lr* = 0.0001) are compared in the table. Since the analysis sets were divided into 70:30 and 80:20 in the study, their ratios are given in the table as a distribution. Table 2 shows the results for N-F-O and N-F-D by applying multiple experimental tests. In addition, Adamax and Stochastic Gradient Descent (SGD) algorithms, commonly used for CNN architectures, were also applied for the optimization algorithm.

Considering the many architectures proposed in the study, the GoogleNet algorithm gave the best results even when different data classes and datasets were considered. It should be noted that the algorithm performance is high, and the test data are prepared separately from the training data. Since the GoogleNet algorithm provides high performance on the datasets, the results of Adamax and SGD optimizers are given in Table 2. Accordingly, for the dataset belonging to the 80:20 distribution of N-F-D classes, the accuracy value of 95.23% with the Adamax optimizer was the highest compared to other data classes. For N-F-O with 70:30 data distribution, the GoogleNet algorithm gave the highest result, 86.61%.

By combining the benefits of the repeated blocks in the GoogleNet algorithm with transfer learning using pre-trained weights, we improve the model’s test performance. Based on the Inception architecture, GoogleNet overcomes a major challenge for a neural network with 22 layers by avoiding feature loss. The performance difference between the Inception-v3 and the GoogleNet classifier is assumed to be due to Inception modules allowing the choice between multiple convolutional filter sizes in each block [26]. The network includes a 1 × 1 convolutional layer with 128 filters and a 70% dropout of the neural network cells to prevent over-memorization [36].

**Figure 3:**
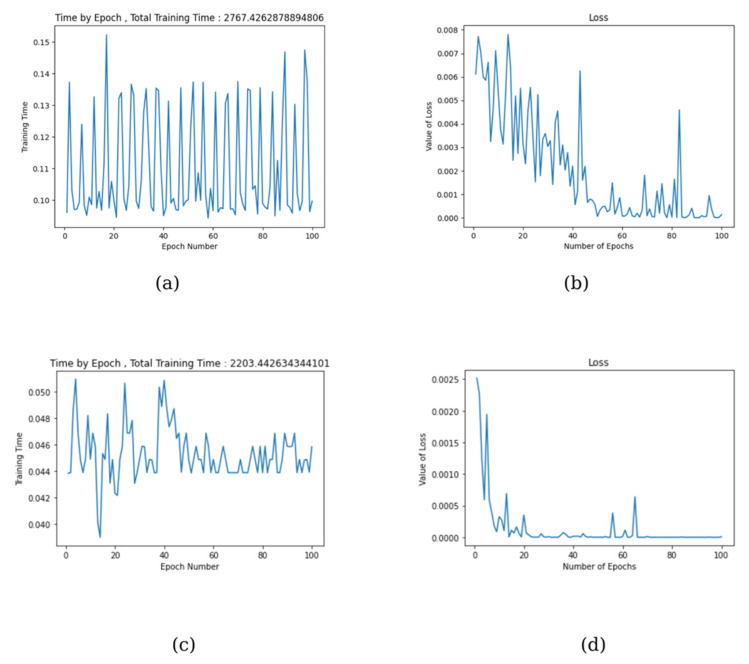
(**a**) DenseNet121 graph of worst total training time-epoch, (**b**) DenseNet121 graph of worst loss-epoch, (**c**) GoogleNet graph of best total training time-epoch, (**d**) GoogleNet graph of best loss-epoch.

Figure 3 shows the performance graphs of the best and worst-performing models of DenseNet and GoogleNet architectures. Accordingly, following the performance findings in Table 1 and Table 2, the 121-layer model of DenseNet shows the worst result with 64.85% accuracy for the 70:30 distribution. In comparison, GoogleNet shows the best result with 95.23% accuracy for the 80:20 distribution. Graphs (a) and (c) show the change in total training time per iteration, while (b) and (d) represent the error (loss) rate of the model per iteration. When graph (b) is analyzed, we see that the loss value has difficulty approaching 0 and shows a very variable structure.

**Figure 4:**
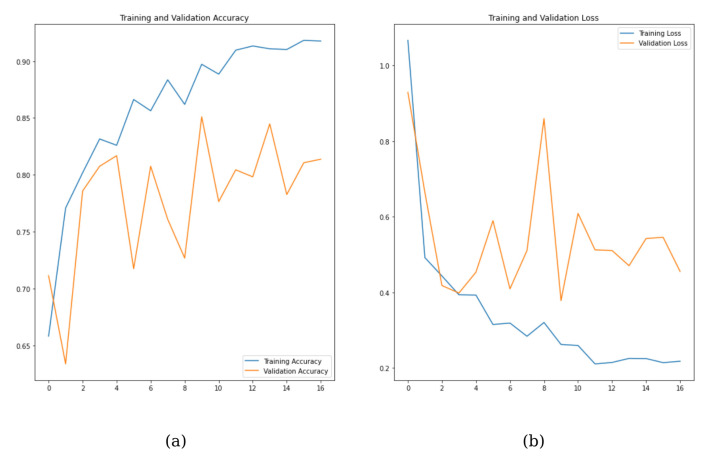
DenseNet121 graph of training and validation results for the epoch. (**a**) DenseNet121 training and validation accuracy values, (**b**) DenseNet121 training and validation loss values.

Figure 4 indicates the training, and validation results up to a certain number of epochs. The blue line (training accuracy) shows a steady rise, reaching about 90%, indicating the model is learning from the data. The orange line (validation accuracy) is more unstable, with frequent ups and downs. While it does generally rise, the possible over-fitting is concerning. The model’s poor generalization capabilities to unseen data are indicated by the widening gap between the training and validation accuracies. Consequently, it can be posited that GoogleNet, as opposed to DenseNet, yields optimal outcomes in this study.

This proves that the model cannot learn enough and is not a very desirable situation. However, in graph (d), the graph starts with a relatively high loss value and gradually tends to approach 0. This is a very common and desirable situation in classification studies. If the training is continued with many iterations, the model becomes over-memorized. For this reason, it was also concluded that further training is inappropriate using enough anti-over-memorization probability.

**Figure 5:**
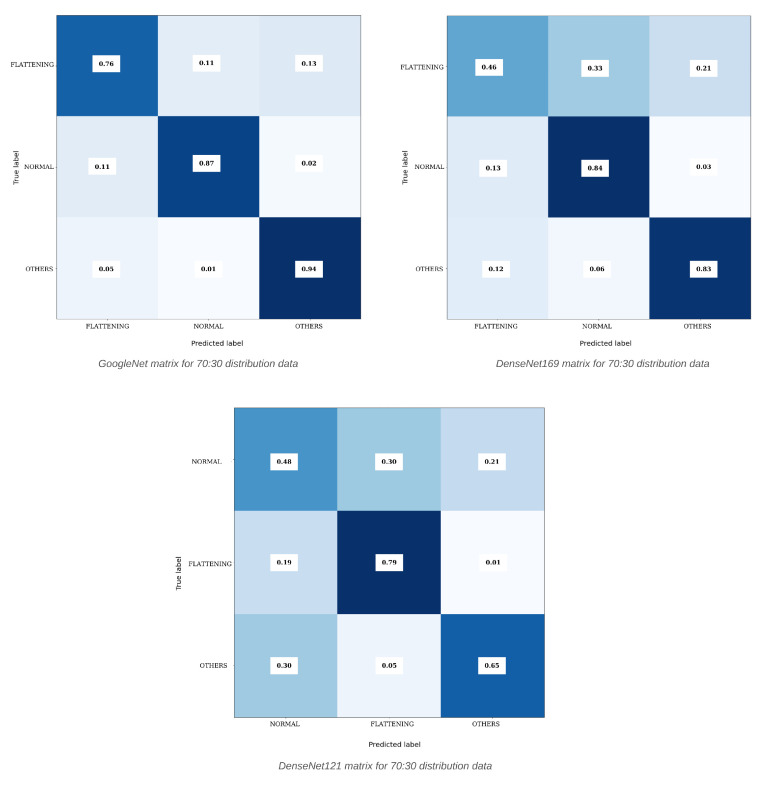
Confusion matrix results obtained with different algorithms for normal-flattening-others classes.

The results of the confusion matrices in Figure 5 are visual evidence for inferring the performance metrics calculated in the equations. When the matrix is examined in detail, the values on the diagonal represent the TP values. TP values indicate that the class to be detected is detected correctly. For this reason, the TP values of each class are expected to be as high as possible. In this case, the relevant TP, TN, FP, and FN values for the performance metrics in the equations are obtained from this matrix and calculated. For the confusion matrix, the horizontal axis shows the predicted labels while the vertical axis contains the actual labels, i.e., oromaxillofacial radiology specialist knowledge. Figure 5 shows the results of the 70:30 dataset distribution for the flattening-normal-others dataset. Here, three confusion matrices are expressed as a percentage ratio. Accordingly, when the first left image is analyzed for the GoogleNet model in the confusion matrix, 76% of correct predictions were made for the “Flattening” class in total. However, 11% of “Normal” and 13% of “Others” were incorrectly predicted. As the highest TP value, 94% correct prediction was made for the “Others” class, but 1% “Normal” and 5% “Flattening” predictions were made.

**Figure 6:**
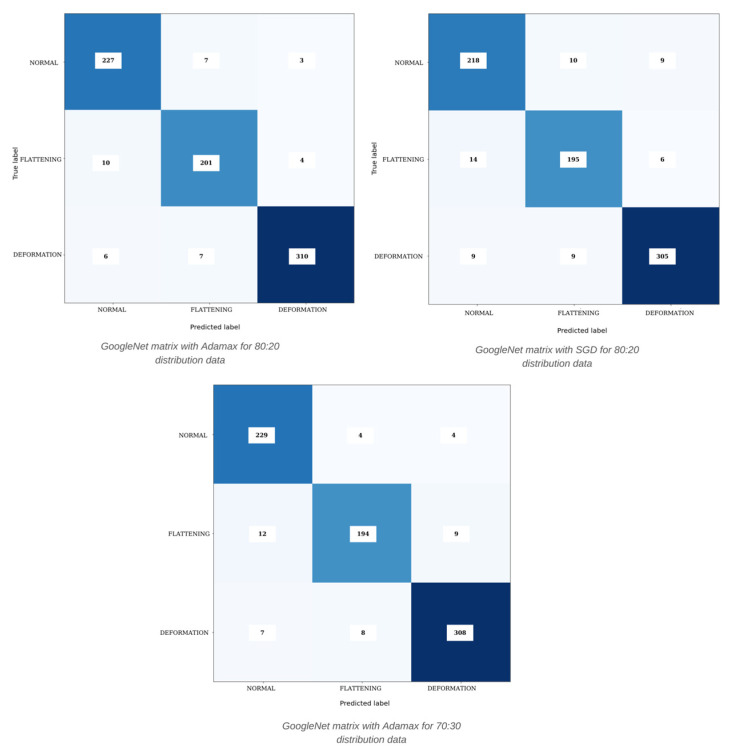
Confusion matrix results were obtained with different algorithms for normal-flattening-deformation classes.

Figure 6 numerically expresses the results of the 70:30 dataset distribution for the normal-flattening-deformation dataset. Considering the measurement results in Table 1 and Table 2, the complexity matrix error values in the figures are appropriate. Accordingly, for the GoogleNet-Adamax optimizer model in the complexity matrix, the “Deformation” class made 310 correct predictions in total. Seven were obtained as “Flattening” and six as “Normal”. When we examine the “Normal” class, while 227 “Normal” predictions were made, 7 “Flattening” and 3 “Deformation” results were obtained. Again, when the matrix is analyzed here, the deformation group was detected with higher success and the flattening and normal groups were detected with relatively lower success.

## 4. Discussion

Mandibular condyle bony changes manifest in a spectrum of variations, ranging from subtle erosions to gross deformities of condylar morphology. A granular classification of these findings furnishes clinicians with valuable insights into disease status. Discordance may exist between clinical symptomatology and radiographic evidence, and inflammatory changes within the temporomandibular joint (TMJ) may, at times, follow a subclinical course.

While ultrasonography and magnetic resonance imaging are the modalities of choice for evaluating the soft tissues of the temporomandibular joint [37,38], conventional radiography and CBCT remain frequently employed for assessing osseous structures.

Panoramic radiography, a conventional radiographic technique utilized for imaging the hard tissues of the temporomandibular joint, offers ready accessibility and cost-effectiveness for general dental practitioners, coupled with a relatively low radiation dose compared to CBCT. However, inherent limitations constrain its utility in TMJ imaging. Panoramic films, acquired with the mandible in a slightly open and protruded position, fail to accurately depict the condyle’s natural position within the glenoid fossa. Furthermore, the superimposition of cranial structures onto the osseous components of the TMJ impedes the detection of subtle alterations [39]. As a result of these shortcomings, panoramic radiographs may prove insufficient for comprehensive condylar and TMJ assessment.

Despite its limitations in accurately evaluating mandibular condyle morphology [40], panoramic radiography possesses the potential to facilitate early diagnosis of TMD due to its low cost, low radiation dose, and routine application [41].

CBCT offers tomographic imaging in all planes, eliminating superimposition and enabling unobstructed visualization of the TMJ region. CBCT demonstrates superior reliability compared to conventional methods in the evaluation of condylar erosions. Consequently, the application of CBCT is advocated as an effective tool for identifying TMJ osteoarthritis [39,42,43,44]. However, CBCT is associated with increased radiation exposure and cost compared to panoramic imaging. Given these considerations, CBCT should be reserved as an advanced imaging modality, with panoramic imaging serving as the preferred initial evaluation to mitigate unnecessary exposure.

These factors impose practical constraints on the widespread adoption of CBCT in clinical settings, thereby impeding researchers’ ability to attain adequate sample sizes for robust investigations.

Accordingly, the present study employed panoramic images to assess the concordance between AI-driven diagnostic outcomes and oromaxillofacial radiology specialist assessments. Achieving alignment between AI diagnostic capabilities and those of an oromaxillofacial radiology specialist would streamline clinical workflows for general dental practitioners.

This study evaluated the agreement between diagnoses rendered by an oromaxillofacial radiology specialist via panoramic images and those derived from AI analysis. Future investigations could explore training AI models using diagnoses informed by both CBCT and panoramic images, enabling a more comprehensive assessment of AI diagnostic accuracy.

Moreover, the implementation of CBCT, an imaging technique that mitigates limitations inherent to panoramic films, may further reduce the likelihood of diagnostic errors by oromaxillofacial radiology specialists, potentially enhancing study outcomes.

Prior literature has predominantly focused on patients with osteoarthritis [31,32,45] and has utilized CBCT imaging [30,45]. The present study sought to classify condylar alterations resulting from both active symptomatic disease (osteoarthritis) and chronic degenerative processes (osteoarthrosis), often used interchangeably, according to features such as flattening, osteophyte formation, erosion, and deformation.

Within this framework, mandibular condyles were categorized into five groups—normal, flattening, osteophyte, erosion, and other—and presented to a CNN for evaluation. The resulting performance was deemed satisfactory.

Notably, the oromaxillofacial radiology specialist incorporated right–left symmetry considerations into condyle classification, particularly in the presence of subtle flattening or pronounced convexity. However, in the context of CNN analysis, condyles were cropped from panoramic images and were not explicitly labeled as right or left. This resulted in a unilateral evaluation of data otherwise interpreted with symmetry, leading the CNN to analyze each condyle independently, disregarding this symmetry.

This study focused on evaluating the performance of CNN models in detecting and classifying mandibular condyle bone changes in panoramic radiographs. As a result of the necessary research and application of the methods, the study showed that CNN-based methods were CNN-based methods demonstrated a high success rate in determining the mandibular condyle bone changes. However, it was concluded that the condyles should be taught dependently with the prediction that the success rate could be further increased. İt is posited that CNN performance could be augmented by segmenting condyles within a single panoramic image and incorporating right–left labeling during training.

Given the limited number of images within the osteophyte and erosion categories, discrete analysis of these conditions was precluded. We anticipate that augmenting the sample size for these groups, while ensuring balanced representation within the overall dataset, would contribute to more robust and accurate findings.

In the present study, a range of deep learning architectures were utilized for the purpose of detecting TMJ-related condylar bone changes using panoramic radiographs. These architectures included AlexNet, VGG16, VGG19, DenseNet121, DenseNet169, DenseNet201, ResNet18, ResNet101, ResNeXt, and GoogleNet. The objective of this research is to illustrate the potential of AI-driven diagnostic support in dentistry and maxillofacial radiology specialists. A distinguishing feature of this study is its rigorous classification strategy. The mandibular condyles were grouped into different categories based on their morphological characteristics, using a novel classification scheme that considers symmetry for images with slight flattening or pronounced convexity. Addressing the challenge of imbalanced datasets is a central tenet of this study, which explores the impact of erosion and osteophyte formation under varied grouping strategies. This exploration aims to enhance the model’s generalizability. Furthermore, model performance was systematically examined using different splits (70:30 and 80:20) to reduce the effects of class imbalance and assess the robustness of the models across different distributions. This methodological approach ensures the reliability and generalizability of the findings. The results show that CNN-based methods achieve a remarkable level of accuracy in detecting mandibular condyle bone changes. These results underscore the effectiveness of deep learning models in providing automated diagnostic assistance that can help clinicians identify patients requiring further intervention. To further validate our labeling methodology, we performed an intra-observer agreement analysis using Cohen’s kappa coefficient, which indicates significant agreement. This step serves to strengthen the reliability of our dataset and classification framework.

Notwithstanding the encouraging outcomes of this study, it is imperative to acknowledge its limitations. First, in our study, panoramic images of individuals over 18 years of age were used to eliminate morphological changes in the condyle during growth and development. However, the AI was trained using panoramic images of individuals over 18 years of age, regardless of age and gender. Future studies can investigate the effects of age and gender differences on AI training. In this way, it will reveal whether age and gender factors have a significant effect on the learning process of AI.

Furthermore, the data utilized in this research are modest in size, which may impede the model’s generalizability. The incorporation of more extensive and varied data could enhance the robustness and precision of the proposed AI models. Notably, the inclusion of erosion and osteophyte classes as other classes and training was necessitated by the limited number of patients. However, the labeling process for annotations and categorization information, a fundamental component of the supervised learning technique, is subject to inter-observer variability and potential bias in ground truth annotations due to the reliance on expert judgment. Ultimately, it is imperative to emphasize that real-world clinical validation remains a crucial step in the development of AI-assisted diagnostic systems. Addressing these limitations in future research will contribute to the development of more robust and clinically applicable AI-assisted diagnostic systems.

## 5. Conclusions

Early and accurate diagnosis of individuals with TMD and referral to specialists for appropriate treatment approaches are critical to the prognosis of the disease. Considering the limited knowledge of general dentists about TMD, the development and integration of AI-supported neural networks into clinical practice could increase the effectiveness of panoramic radiographs in TMJ assessment. It is believed that this approach could provide significant advantages to both general dentists and patients by providing AI-based support in the diagnosis and treatment processes.

As a result, this study achieved a high success rate by using CNNs to determine the mandibular condyle bone changes. We think that identifying the changes in this region, which are difficult to evaluate on panoramic images, with CNN will make it easier for clinicians to refer patients to physicians who are experts in this field.

## Figures and Tables

**Table 1:** Performance results for normal, flattening, and other data classes with a certain distribution range.

Model	Distribution	Learning Rate	Optimizer	Accuracy	Precision	Recall	F1-Score
DenseNet121	70:30	0.001	Adamax	64.85%	64.45%	64.22%	63.88%
ResNet18	70:30	0.0001	Adamax	69.23%	65.52%	66.41%	65.65%
AlexNet	70:30	0.0001	Adamax	70.71%	68.74%	68.55%	68.47%
DenseNet169	70:30	0.0001	Adamax	73.43%	71.05%	71.04%	70.66%
GoogleNet	70:30	0.0001	Adamax	86.61%	85.92%	85.60%	85.73%

**Table 2:** Comparative performance results for normal, flattening, others and normal, flattening, deformation data classes with different distribution ranges (N: Normal, F: Flattening, O: Others, D: Deformation).

Model	Distribution	Dataset	Optimizer	Accuracy	Precision	Recall	F1-Score
DenseNet169	70:30	N-F-O	Adamax	73.43%	73.29%	73.12%	73.20%
GoogleNet	70:30	N-F-O	Adamax	86.61%	85.92%	85.60%	85.73%
GoogleNet	80:20	N-F-D	SGD	92.65%	92.29%	92.36%	92.33%
ResNeXt	80:20	N-F-D	Adamax	94.32%	94.15%	94.07%	94.08%
GoogleNet	80:20	N-F-D	Adamax	95.23%	94.89%	95.08%	94.98%

## Data Availability

The original contributions presented in this study are included in this article, and further inquiries can be directed to the corresponding author.

## References

[1] Sritara S., Tsutsumi M., Fukino K., Matsumoto Y., Ono T., Akita K. (2021). Evaluating the morphological features of the lateral pterygoid insertion into the medial surface of the condylar process. Clin. Exp. Dent. Res..

[2] Suh M., Park S., Kim Y.-K., Yun P.-Y., Lee W. (2018). 18F-NaF PET/CT for the evaluation of temporomandibular joint disorder. Clin. Radiol..

[3] Minervini G., D’Amico C., Cicciù M., Fiorillo L. (2023). Temporomandibular joint disk displacement: Etiology, diagnosis, imaging, and therapeutic approaches. J. Craniofacial Surg..

[4] Crincoli V., Anelli M.G., Quercia E., Piancino M.G., Di Comite M. (2019). Temporomandibular disorders and oral features in early rheumatoid arthritis patients: An observational study. Int. J. Med. Sci..

[5] Schiffman E., Ohrbach R., Truelove E., Look J., Anderson G., Goulet J.-P., List T., Svensson P., Gonzalez Y., Lobbezoo F. (2014). Diagnostic criteria for temporomandibular disorders (DC/TMD) for clinical and research applications: Recommendations of the International RDC/TMD Consortium Network and Orofacial Pain Special Interest Group. J. Oral Facial Pain Headache.

[6] Zieliński G., Pająk-Zielińska B., Ginszt M. (2024). A meta-analysis of the global prevalence of temporomandibular disorders. J. Clin. Med..

[7] Bliddal H. (2020). Definition, pathology and pathogenesis of osteoarthritis. Ugeskr. Laeger.

[8] Cardoneanu A., Macovei L.A., Burlui A.M., Mihai I.R., Bratoiu I., Rezus I.I., Richter P., Tamba B.-I., Rezus E. (2022). Temporomandibular joint osteoarthritis: Pathogenic mechanisms involving the cartilage and subchondral bone, and potential therapeutic strategies for joint regeneration. Int. J. Mol. Sci..

[9] Mureșanu S., Hedeșiu M., Iacob L., Eftimie R., Olariu E., Dinu C., Jacobs R., Team Project Group (2024). Automating Dental Condition Detection on Panoramic Radiographs: Challenges, Pitfalls, and Opportunities. Diagnostics.

[10] ArunKumar G. (2021). Bone changes in condyles of asymptomatic temperomandibular joints & its correlation with age, gender and occlusal condition; a digital panoramic study. J. Dent. Health Oral Disord. Ther..

[11] Görürgöz C., İçen M., Kurt M., Aksoy S., Bakırarar B., Rozylo-Kalinowska I., Orhan K. (2023). Degenerative changes of the mandibular condyle in relation to the temporomandibular joint space, gender and age: A multicenter CBCT study. Dent. Med. Probl..

[12] Schroder Â.G.D., Gonçalves F.M., Germiniani J.d.S., Schroder L.D., Porporatti A.L., Zeigelboim B.S., de Araujo C.M., Santos R.S., Stechman-Neto J. (2023). Diagnosis of TMJ degenerative diseases by panoramic radiography: Is it possible? A systematic review and meta-analysis. Clin. Oral Investig..

[13] Valesan L.F., Da-Cas C.D., Réus J.C., Denardin A.C.S., Garanhani R.R., Bonotto D., Januzzi E., de Souza B.D.M. (2021). Prevalence of temporomandibular joint disorders: A systematic review and meta-analysis. Clin. Oral Investig..

[14] Mani F.M., Sivasubramanian S.S. (2016). A study of temporomandibular joint osteoarthritis using computed tomographic imaging. Biomed. J..

[15] Osiewicz M., Kojat P., Gut M., Kazibudzka Z., Pytko-Polończyk J. (2020). Self-Perceived Dentists’ Knowledge of Temporomandibular Disorders in Krakow: A Pilot Study. Pain Res. Manag..

[16] Mozhdeh M., Caroccia F., Moscagiuri F., Festa F., D’Attilio M. (2020). Evaluation of knowledge among dentists on symptoms and treatments of temporomandibular disorders in Italy. Int. J. Environ. Res. Public Health.

[17] Schwendicke F., Golla T., Dreher M., Krois J. (2019). Convolutional neural networks for dental image diagnostics: A scoping review. J. Dent..

[18] Shafi I., Fatima A., Afzal H., Díez I.D.L.T., Lipari V., Breñosa J., Ashraf I. (2023). A Comprehensive Review of Recent Advances in Artificial Intelligence for Dentistry E-Health. Diagnostics.

[19] Özbay Y., Kazangirler B.Y., Özcan C., Pekince A. (2024). Detection of the separated endodontic instrument on periapical radiographs using a deep learning-based convolutional neural network algorithm. Aust. Endod. J..

[20] Bayrakdar I.S., Orhan K., Akarsu S., Çelik Ö., Atasoy S., Pekince A., Yasa Y., Bilgir E., Sağlam H., Aslan A.F. (2022). Deep-learning approach for caries detection and segmentation on dental bitewing radiographs. Oral Radiol..

[21] Kreiner M., Viloria J. (2022). A novel artificial neural network for the diagnosis of orofacial pain and temporomandibular disorders. J. Oral Rehabil..

[22] Chauhan R., Ghanshala K.K., Joshi R. (2018). Convolutional neural network (CNN) for image detection and recognition. Proceedings of the 2018 First International Conference on Secure Cyber Computing and Communication (ICSCCC).

[23] Ozsari S., Güzel M.S., Yılmaz D., Kamburoğlu K. (2023). A Comprehensive Review of Artificial Intelligence Based Algorithms Regarding Temporomandibular Joint Related Diseases. Diagnostics.

[24] Zhu Z., Lin K., Jain A.K., Zhou J. (2023). Transfer learning in deep reinforcement learning: A survey. IEEE Trans. Pattern Anal. Mach. Intell..

[25] Shaha M., Pawar M. (2018). Transfer learning for image classification. Proceedings of the 2018 Second International Conference on Electronics, Communication and Aerospace Technology (ICECA).

[26] Farook T.H., Dudley J. (2023). Automation and deep (machine) learning in temporomandibular joint disorder radiomics: A systematic review. J. Oral Rehabil..

[27] Yazici İ., Shayea I., Din J. (2023). A survey of applications of artificial intelligence and machine learning in future mobile networks-enabled systems. Eng. Sci. Technol. Int. J..

[28] Nishiyama M., Ishibashi K., Ariji Y., Fukuda M., Nishiyama W., Umemura M., Katsumata A., Fujita H., Ariji E. (2021). Performance of deep learning models constructed using panoramic radiographs from two hospitals to diagnose fractures of the mandibular condyle. Dentomaxillofacial Radiol..

[29] Ahn Y., Hwang J.J., Jung Y.-H., Jeong T., Shin J. (2021). Automated mesiodens classification system using deep learning on panoramic radiographs of children. Diagnostics.

[30] Lee K., Kwak H., Oh J., Jha N., Kim Y., Kim W., Baik U., Ryu J. (2020). Automated detection of TMJ osteoarthritis based on artificial intelligence. J. Dent. Res..

[31] Choi E., Kim D., Lee J.-Y., Park H.-K. (2021). Artificial intelligence in detecting temporomandibular joint osteoarthritis on orthopantomogram. Sci. Rep..

[32] Jung W., Lee K., Suh B., Seok H., Lee D. (2023). Deep learning for osteoarthritis classification in temporomandibular joint. Oral Dis..

[33] Hegde S., Praveen B., Shetty S.R. (2013). Morphological and radiological variations of mandibular condyles in health and diseases: A systematic review. Dentistry.

[34] Mallya S., Lam E. (2018). White and Pharoah’s Oral Radiology: Principles and Interpretation.

[35] Tekin B.Y., Ozcan C., Pekince A., Yasa Y. (2022). An enhanced tooth segmentation and numbering according to FDI notation in bitewing radiographs. Comput. Biol. Med..

[36] Szegedy C., Liu W., Jia Y., Sermanet P., Reed S., Anguelov D., Erhan D., Vanhoucke V., Rabinovich A. Going deeper with convolutions. Proceedings of the IEEE Conference on Computer Vision and Pattern Recognition.

[37] Pekince K.A., Caglayan F., Pekince A. (2020). Imaging of masseter muscle spasms by ultrasonography: A preliminary study. Oral Radiol..

[38] Pekince K.A., Çağlayan F., Pekince A. (2020). The efficacy and limitations of USI for diagnosing TMJ internal derangements. Oral Radiol..

[39] Walewski L.Â., de Souza Tolentino E., Yamashita F.C., Iwaki L.C.V., da Silva M.C. (2019). Cone beam computed tomography study of osteoarthritic alterations in the osseous components of temporomandibular joints in asymptomatic patients according to skeletal pattern, gender, and age. Oral Surg. Oral Med. Oral Pathol. Oral Radiol..

[40] Schmitter M., Gabbert O., Ohlmann B., Hassel A., Wolff D., Rammelsberg P., Kress B. (2006). Assessment of the reliability and validity of panoramic imaging for assessment of mandibular condyle morphology using both MRI and clinical examination as the gold standard. Oral Surg. Oral Med. Oral Pathol. Oral Radiol. Endodontology.

[41] Singh B., Kumar N.R., Balan A., Nishan M., Haris P.S., Jinisha M., Denny C.D. (2020). Evaluation of normal morphology of mandibular condyle: A radiographic survey. J. Clin. Imaging Sci..

[42] Abrahamsson A.-K., Kristensen M., Arvidsson L.Z., Kvien T.K., Larheim T.A., Haugen I.K. (2017). Frequency of temporomandibular joint osteoarthritis and related symptoms in a hand osteoarthritis cohort. Osteoarthr. Cartil..

[43] Sonnesen L., Petersson A., Wiese M., Jensen K.E., Svanholt P., Bakke M. (2017). Osseous osteoarthritic-like changes and joint mobility of the temporomandibular joints and upper cervical spine: Is there a relation?. Oral Surg. Oral Med. Oral Pathol. Oral Radiol..

[44] Dumbuya A., Gomes A.F., Marchini L., Zeng E., Comnick C.L., Melo S.L.S. (2020). Bone changes in the temporomandibular joints of older adults: A cone-beam computed tomography study. Spec. Care Dent..

[45] de Dumast P., Mirabel C., Cevidanes L., Ruellas A., Yatabe M., Ioshida M., Ribera N.T., Michoud L., Gomes L., Huang C. (2018). A web-based system for neural network based classification in temporomandibular joint osteoarthritis. Comput. Med. Imaging Graph..

